# The Detection of Wound Infection by Ion Mobility Chemical Analysis

**DOI:** 10.3390/bios10030019

**Published:** 2020-02-29

**Authors:** Emma Daulton, Alfian Wicaksono, Janak Bechar, James A. Covington, Joseph Hardwicke

**Affiliations:** 1 School of Engineering, University of Warwick, Coventry CV4 7AL, UK; E.Daulton@warwick.ac.uk (E.D.); A.Wicaksono@warwick.ac.uk (A.W.); 2 Warwick Medical School, University of Warwick, Medical School Building, Coventry CV4 7HL, UK; Janak.Bechar@warwick.ac.uk (J.B.); J.Hardwicke@warwick.ac.uk (J.H.); 3 Department of Plastic Surgery, University Hospitals of Coventry and Warwickshire NHS Trust, Clifford Bridge Road, Coventry, CV2 2DX, UK

**Keywords:** wound infection, gas analysis, diagnosis, VOC, GC-IMS

## Abstract

Surgical site infection represents a large burden of care in the National Health Service. Current methods for diagnosis include a subjective clinical assessment and wound swab culture that may take several days to return a result. Both techniques are potentially unreliable and result in delays in using targeted antibiotics. Volatile organic compounds (VOCs) are produced by micro-organisms such as those present in an infected wound. This study describes the use of a device to differentiate VOCs produced by an infected wound vs. colonised wound. Malodourous wound dressings were collected from patients, these were a mix of post-operative wounds and vascular leg ulcers. Wound microbiology swabs were taken and antibiotics commenced as clinically appropriate. A control group of soiled, but not malodorous wound dressings were collected from patients who had a split skin graft (SSG) donor site. The analyser used was a G.A.S. GC-IMS. The results from the samples had a sensitivity of 100% and a specificity of 88%, with a positive predictive value of 90%. An area under the curve (AUC) of 91% demonstrates an excellent ability to discriminate those with an infected wound from those without. VOC detection using GC-IMS has the potential to serve as a diagnostic tool for the differentiation of infected and non-infected wounds and facilitate the treatment of wound infections that is cost effective, non-invasive, acceptable to patients, portable, and reliable.

## 1. Introduction

The skin is the largest organ in the human body and provides a critically important barrier to the external environment, allowing homeostasis and protecting against infection [1]. The skin is also important in temperature control and allows for the sensing of the external environment. Volatile organic compounds (VOCs) originate from skin structures (such as eccrine, apocrine, and sebaceous glands) as well as from skin commensal organisms [2,3]. VOCs are thought to be by-products of normal metabolic pathways [4]. Their production is usually dependent on species, strain, growth phase, pH, nutrients, and co-existing environmental conditions [5]. VOCs represent a heterogenous cohort of chemicals and include ketones, alcohols, esters, and sulphur compounds amongst others [6]. The five most common VOCs from human skin are 6-methyl-5-hepten-2-one, nonanal, decanal, geranylacetine and (E)-2- nonenal [7]. VOCs are potential biomarkers of occult disease. To date, these VOC patterns have been identified in patients with melanoma using electronic noses [8].

Chronic wounds are a significant financial burden to the National Health Service, with expenditure exceeding £1 billion per year [9]. A total of 50 million surgical procedures are performed in the United States, with surgical site infection incidence being as high as 5% with a high mortality association [10]. Open wounds resulting from trauma or surgery are of particular importance to clinicians because the loss of the primary barrier of skin makes them much more susceptible to infection. At present, the standard of care for large open wounds is to assess them primarily by their clinical appearance in addition to the examination of the host response to a possible infection. Wounds may also have an offensive odour resulting from either specific bacterial colonisation or from tissue necrosis. If there is a clinical suspicion of wound and/or surrounding skin infection (redness, swelling, heat, pain), empirical antibiotics therapy is commenced. However, there is no single objective point of care (POC) method of distinguishing between an infected malodorous wound and a non-infected colonized wound, and there are no clinical signs or immediate POC tests available to give a causative microorganism.

For infected wounds, the current standard of care is empirical treatment with antibiotics in addition to specialist wound care. Antimicrobial therapy is commenced immediately based on a “best guess” approach to the common causative organism. The wound is swabbed, or tissue is taken for microscopy and culture, which typically takes between 48–72 h. This helps determine bacterial sensitivity or resistance to the empirical treatment. However, swab results may be misleading, as clinical microbiology laboratories use culture methods that select for planktonic bacteria or are not always suitable for anaerobic species. A wound culture also might not capture bacteria protected within a biofilm, so the result can be inconclusive [11,12,13].

The disadvantage with this current methodology is two-fold. First, patients with a truly infected wound may receive incorrect antibiotic therapy, resulting in the worsening of infection and increased hospital stay. Second, empirical antibiotics, either due to over-prescription or incorrect provision for colonised wounds, remain a major risk factor for antimicrobial resistance (AMR). In over prescription, the rise of easily transmissible genetic elements encoding resistance to last line antimicrobials raises the real possibility of a post-antibiotic era. This demonstrates a pressing need for a novel, fast and reliable POC test to guide correct antibiotic prescription in primary and secondary care.

A potential solution to this is to detect and monitor the odours emanating from wounds and the dressings in contact with the wound. These gas phase biomarkers can be detected with a range of different analytical instrumentation. To this end, researchers have previously shown that high-end analytical instrumentation, such as GC-MS (gas chromatograph mass spectrometers—the gold standard for this purpose) is able to detect and identify infections [14]. However, such instruments are large, expensive, bulky, and require highly trained staff to use. For this reason, a number of researchers have used electronic noses—instruments that attempt to mimic the biological olfactory system—as a means of detecting these biomarkers [15]. Though they show considerable promise, they are unable to detect individual chemical components in a complex mixture. Furthermore, many of these instruments are formed from arrays of metal-oxide gas sensors that drift over time. An alternative technology is ion mobility spectrometry (IMS), now used extensively in security applications. These instruments have high sensitivity, low drift, and good levels of selectivity. For this reason, they have found favour in the medical diagnostic arena [16,17,18].

In this paper, we report on the first use of ion mobility spectrometry in the detection of volatiles from infected wounds and compare this to normal healthy controls as a potential means of monitoring for wound infection.

## 2. Materials and Methods

### 2.1. Patient Recruitment

Malodourous wound dressings were collected from patients recruited from plastic surgery, vascular surgery, and diabetic dressings clinics at University Hospitals of Coventry and Warwickshire NHS Trust (UHCW) as part of the Olfactory Biosensors for Offensive Wound Evaluation (OBOWE) pilot observational study. These were a mix of post-operative wounds and arterial and/or venous leg ulcers. Wound microbiology swabs were taken as the current standard of care, antibiotics commenced as clinically appropriate, and the wounds were redressed as normal. A control group of soiled, but not malodorous, wound dressings were collected from patients who had a split skin graft (SSG) donor site at the routine 14-day post-op donor site check. Wound site, size, dressing, and duration were variable in the study group, but for the control this was always a lateral thigh (right or left) SSG donor with a consistent dressing—gauze and acrylic adhesive (polyester elastic nonwoven fabric).

Strict inclusion and exclusion criteria were applied for patient recruitment. All patients had to be 18 years or above. For a patient in the study group, they had to have a clinically malodourous dressing that was a non-antimicrobial (e.g., paraffin tulle gras, cellulose acetate fabric with petrolatum emulsion, polyurethane foam), a wound of any size, causation, or duration. The exclusion criteria for the study group was any type of antimicrobial dressing, for example, silver, iodine, chlorhexidine, honey, any suspected active cutaneous malignancy, and a recent course of oral or intravenous antibiotics (within the last 14 days). For the control group, the inclusion criteria were split skin graft donor sites that were clinically non-malodorous. The exclusion criteria for the control group was any malodourous or clinically infected wound. Wound dressings were collected under NHS Research Ethics Service approval at UHCW (Arden Tissue Bank NRES generic ethical approval reference 12/SC/0526). Dressings were placed in a collection bag and transported to the Arden Tissue Bank where they were snap frozen and stored at −80 °C. As the data was anonymized only the age and sex of each patient can be reported. Dressings were from 19 males and 5 females between the ages of 27 to 90.

### 2.2. Chemical Analyzer

The chemical analyser used in this study was a G.A.S. GC-IMS (Dortmund, Germany), which is used for a range of industrial applications. In use, the headspace of the swab (i.e., the odours emanating from the sample) are injected into the instrument to identify the measure VOCs. The instrument is formed of a gas chromatography (GC) column (FS-SE-54-CB-1, 30 m × 0.44 mm (OD) × 0.32 mm (ID), CS Chromatographie Service GmbH, Langerwehe, Germany), followed by a drift tube ion mobility spectrometer. Thus, the GC component separates the chemical components based on the interaction between the molecules and the stationary phase coating on the wall of the column. These molecules are then ionized (in this case by a tritium source) and are moved along the drift tube by an electric field. A buffer gas (in our case pure nitrogen) flows in the opposite direction to the ions, resulting in ion/nitrogen collisions. Depending on the mass and charge of the ion, they are selectively slowed, providing some level of molecular separation. Thus, the unit provides the retention and drift times of molecules on test. We chose to use this instrument over more traditional GC-MS because the basic sensitivity of GC-IMS is much higher than GC-MS, it uses nitrogen/air as the carrier gas (thus no expensive carrier gas, such as helium), it has a lower purchase/sample test cost, and it has a much smaller form factor, making it applicable for use in a ward (dimensions: 45 × 50 × 20 cm; mass: 20 kg).

### 2.3. Sample Testing

The samples were frozen at −80 °C and transported on dry ice to Warwick University. The samples were then thawed overnight in a laboratory fridge (4 °C) to minimize chemical loss. A section of the dressing was then removed and transferred to a 20 mL glass vial and sealed with a crimp top lid. Once completed, 5 mL of headspace (the air above the swab) was collected using a needle and syringe and injected directly into the GC-IMS. The GC-IMS was operated using the following settings: E1 flow 150 mL/min (for the drift tube IMS), E2 flow 20 mL/min (for the GC column), temperature 1 45 °C (IMS), temperature 280 °C (GC column), temperature 370 °C (sample port). The valve responsible for allowing the sample to flow into the GC (V1) was opened for a total of 6 s at 20 mL/min (flow E2), allowing 2 mL of the sample headspace to be used for analysis.

### 2.4. Data Analysis

**Figure 1:**
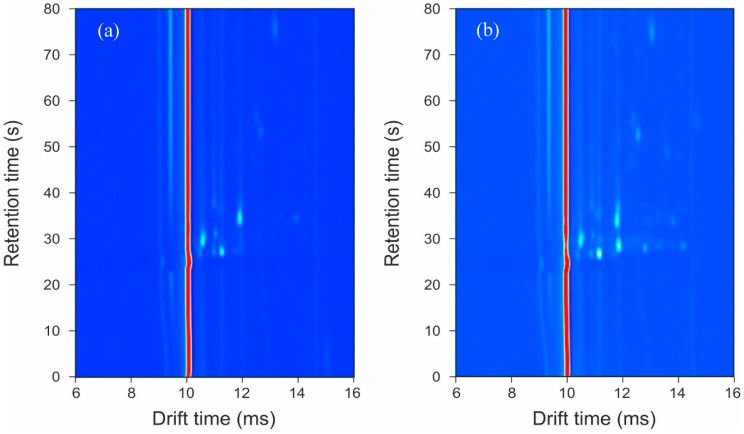
Typical output plots from the GC-IMS instrument: (**a**) control sample; (**b**) infected sample.

A typical output from the GC-IMS for a control and an infected sample is shown in Figure 1. In this figure, the background is represented in dark blue with the red and lighter blue areas showing that the instrument is detecting molecules. The intensity of the peak (with red being the highest intensity) represents the number of ions, and thus the chemical detected. The circular spots are chemicals being detected/separated. The red line is the reactant ion peak (RIP) and shows the output of the instrument when there are no chemicals present. The output shows how most of the chemical information from a sample is in a condensed region towards the bottom of the *y*-axis. It is this region that is cropped and used for data analysis.

The GC-IMS output in Figure 1 clearly shows that extensive chemical information is being collected from the samples. In addition, a visual inspection already shows differences between the sample groups, which are neither associated with the dressing nor the person’s skin. This chemical information can be found to the right of the RIP.

The GC-IMS creates very high dimensional datasets, totaling 11 million data points per sample. Though GC-IMS has a high dimensionality, not all 11 million data points contain relevant sample information. The first step in the data analysis involved a pre-processing stage to extract only the information necessary to build and train the diagnostic model. The data is first cropped to only include useful sample information. Here, the central section of the data is removed, where all the chemical information lies. The dimensions of this crop are chosen based on visual inspection of all of the data and exactly the same crop value was used for each sample. Then, a threshold was set to further remove areas containing no useful chemical information, thus removing the background. This is the output of the instrument when no chemicals are present and represents the ‘noise’ of the system. These steps reduce the dimensionality to around 10,000 data points of non-zero values. The remaining data is then analysed using our previously developed pipeline [19,20,21]. In brief, to reduce the problem of over classification, a 10-fold cross validation was used, where the data was split into 90% training set and 10% test set. For feature selection, a Wilcoxon rank sum was used to calculate p-values in the training set to identify which features are optimum for infection prediction and the smallest 20 used for classification. Two classification algorithms were applied, sparse logistic regression and random forest, which we have previously used on such clinical studies. The data analysis pipeline was written in ‘R’ (version 3.6.1). A receiver operator characteristic (ROC) curve was created to predict area under curve (AUC), sensitivity, specificity, positive predictive value (PPV), negative predictive value (NPV), and p-values.

## 3. Results

**Figure 2:**
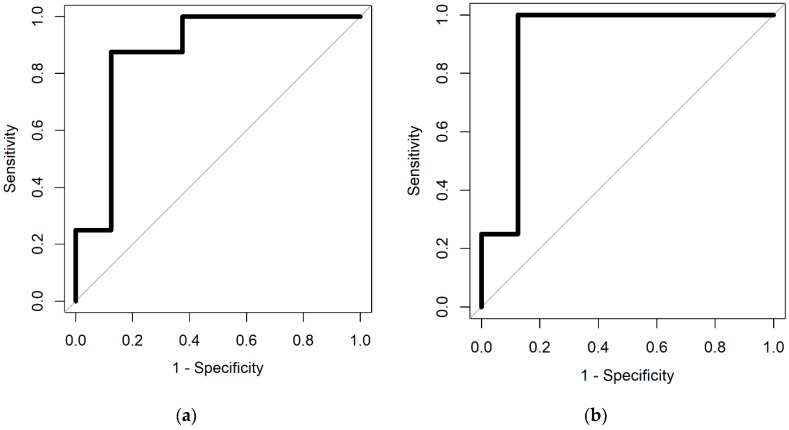
Receiver operator characteristic (ROC) curves from (**a**) spare logistic regression and (**b**) random forest classifiers.

The results of the statistical analysis, described in Section 2.4, are given in Table 1. Figure 2 shows the ROC curves for the two different classifiers.

**Figure 3:**
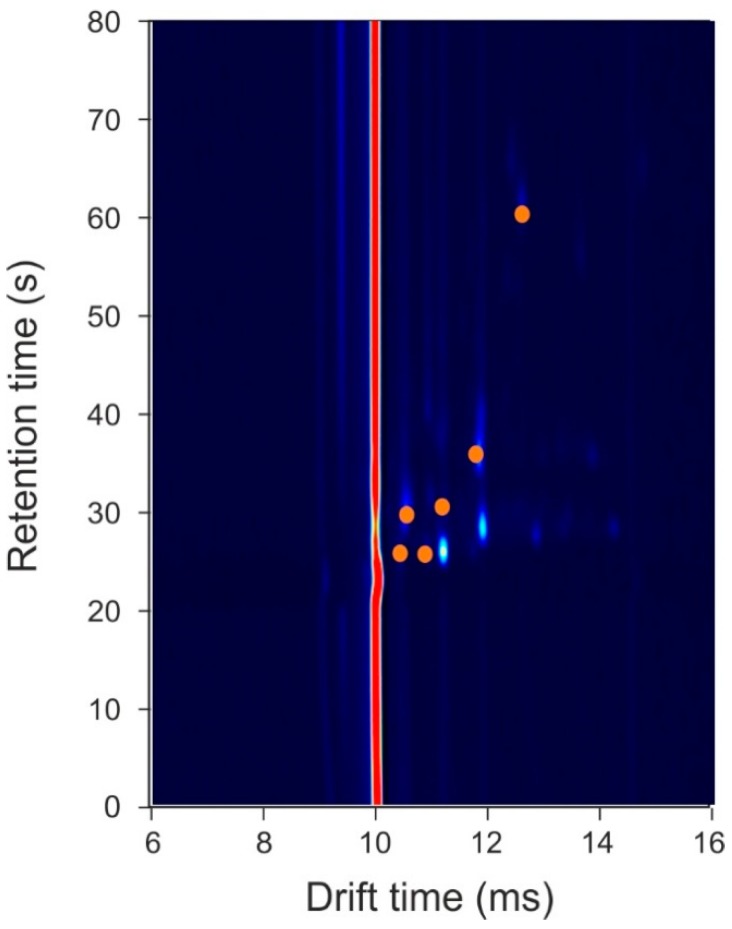
Feature locations that hold discriminatory information. Orange circles represent locations where data points were used for the statistical analysis. Note, the background colour has been darkened to make the chemical locations stand out.

The results demonstrate high sensitivity and specificity, indicating that there are significant differences between the VOC profiles of infected wound samples, compared to those not infected. Using our statistical approach, we can identify the feature locations (specific VOC peaks, highlighted in Figure 3), which hold discriminatory information.

Due to the small sample size, we did not attempt to separate the different wound infection types or causative organisms. In the study group, the bacterial species that were cultured were Staphylococcus aureus (n = 4), Pseudomonas aeruginosa (n = 1), Serratia marcescens (n = 1), mixed faecal flora (n = 2), mixed skin flora (n = 2), and mixed coliforms (n = 2). One infection with Candida albicans was also recorded. In the control group, the bacterial species were Staphylococcus aureus (n = 1), mixed skin flora (n = 5), mixed coliforms (n = 3). Many results were polymicrobial and some showed no growth after 48 h. A summary of the different bacterial species and the number of wound infections are shown Table 2.

## 4. Discussion

To the best of our knowledge, this was the first study to use GC-IMS technology for the analysis of wound samples. Here, we compared traditional culture methods of swabs with the analysis of the VOCs emanating from a range of wound infections and clean samples. Our results indicate that there are a number of chemicals associated with the infection. These chemicals are likely to be the result of the metabolic activity of the bacteria itself, which differs both from the odours from the skin (control patients) and from the swab due to them being present in samples of differing gauze material, or through the interaction of the bacteria with the host or the swab. The technology used in this study (GC-IMS), to analyse VOCs from the swabs, is rapid (under 10-min) and has the potential to be used in a ward, outpatient, or community setting since it does not require any additional services beyond a power supply. The results of this pilot study will inform the streamlining of the analysis as well as the design and build of equipment to investigate this specific region of interest. However, the study has a number of limitations. First, the sample size used here for this pilot study was small and therefore a much larger study is required to fully understand the potential of this approach. Second, due to the small sample size, we did not attempt to separate the different wound infection types. The results indicate that there are common odours across the different infections. Furthermore, using our approach, we were unable to identify the specific chemical biomarkers associated with the infections. The discriminatory features, highlighted in Figure 3, are small/light molecules, since they appear in the output plot at relatively low retention times. These lighter molecules could be produced as a result of metabolic activity of the bacteria present in the wound.

To date, there are no articles in the peer reviewed literature describing the role of VOC pattern detection directly from surgical site infections, as opposed to those from cultures of swabs. For example, Sun et al. collected samples from swabs from infected wound sites, cultured the swabs in broth, and then analysed the samples by FAIMS (Field Asymmetric Ion Mobility Spectrometry) and showed a high accuracy in detecting an infected sample [16]. Furthermore, studies have started to describe the use of VOCs in cutaneous wounds. Parry et al. investigated the use of an electronic nose to distinguish between an uninfected venous ulcer and a beta-haemolytic streptococci infection in venous ulcers [22]. The authors interrogated 24 chronic venous ulcers and found that the electronic nose demonstrated clear differences in 20 sensor aroma profiles of non-infected ulcers and those ulcers with culture proven beta-haemolytic streptococci infection to statistical significance. Thomas et al. used gas chromatography ion trap mass spectrometry on VOCs from five patients with chronic lower limb wounds [23]. The authors used a polydimethylsilicone membrane to attain VOCs sampled from the wound, the boundary region of the wound, and normal skin, showing that each area had a unique profile. Thomas et al. identified six compounds that may be responsible for these differences: 1-(1-methyethoxy) 2-propanol; dimethyl disulfide; 3-carene; 2-ethyl-1- hexanol; 3,5-bis(1,1-dimethylethyl)-phenol; and butylated hydroxytoluene. However, these compounds are also found in skin creams and gels and may demonstrate another limitation of using VOCs.

Another novel application of skin VOCs was demonstrated by Dina et al. [24]. The authors compared VOCs from compressed and non-compressed skin using GC-MS. Their aim was to elucidate the pattern of VOCs from skin with compressive forces applied that were at risk of developing pressure wounds. The authors found that compressed skin emitted a differing pattern of VOCs compared to non-compressed skin. This may point to the future in being able to non-invasively detect the risk of developing wounds. 

VOCs emitted from micro-organisms causing wound infection are diverse [25]. There are many VOCs that are shared amongst micro-organisms, but identifying each organism based on VOC profiles at the point of care may reveal further benefits in targeted antimicrobial therapy. Our pilot study was able to differentiate between an infected and colonised wound but was unable to differentiate between different micro-organisms causing infection. However, studies looking at VOCs of the same organism found differing profiles, which may be explained by differing sampling and analysis techniques [19]. Bacteria such as Pseudomonas aeruginosa and Escherichia coli are associated with a wide spectrum of VOCs [22]. This, however, illustrates the difficulty in bacteria VOC characterisation. Furthermore, VOCs examined both in our study and others have been assumed to be collected from bacteria in a planktonic phase. The profile of VOCs may thus be different from bacteria encapsulated within a biofilm, as the biofilm may have unique VOC characteristics [25]. In the clinical setting, the control of external factors may also be challenging as minimizing exogenous VOCs in the clinical setting could be challenging. 

To date, the FDA has approved devices for VOC detection in the diagnosis of asthma [26], H. pylori infection [27], and heart transplant rejection [28]. Given that VOCs can provide a non-invasive avenue for diagnosis at the point of care, the scope for using VOCs in the diagnosis of surgical site infections is an exciting new development in patient care. However, further in-vivo and in-vitro work, along with robust clinical studies, still need to be performed to identify discrete patterns of common microbe VOCs attributed to the aetiology of surgical site infections. VOC detection using GC-IMS has the potential to be a diagnostic tool that is cost effective, non-invasive, acceptable to patients, portable, and reliable.

## 5. Conclusions

The detection and monitoring of infected wounds remains a clinical issue. Here, we report on the use of a commercial GC-IMS to detect the odours emanating from infected wounds and colonised wound dressings after surgery. In total, 19 different samples were tested, from a range of infected and non-infected dressings collected from patients. The results indicate a sensitivity reaching 100% and a specificity of 88%. These signals can be visually seen on the output of the GC-IMS instrument. We believe this is the first paper to report on the use of GC-IMS for this purpose directly from swabs rather than from culture. In the future, we will look at attempting to identify the specific VOCs involved and undertaking a larger study to evaluate if we are able to distinguish between specific bacterial groups.

## Figures and Tables

**Table 1:** Statistical analysis of GC-IMS data.

	Sparse Logistic Regression		Random Forest	
Parameter	Value	95% C.I.	Value	95% C.I.
**Area Under the Curve**	0.88	0.68–1	0.91	0.71–1
**Sensitivity**	0.89	0.47–1	1	0.63–1
**Specificity**	0.87	0.47–1	0.88	0.47–1
**PPV**	0.9		0.9	
**NPV**	0.9		1	
**p-value**	0.0047		0.0027	

**Table 2:** Cultured Bacterial species from the Study Group and Control samples.

Bacterial Species	Number of Confirmed Bacteria Present in Study Group	Number of Confirmed Bacteria Present in Control group
Staphylococcus aureus	4	1
Pseudomonas aeruginosa	1	0
Serratia marcescens	1	0
Mixed faecal flora	2	0
Mixed skin flora	2	5
Mixed coliforms	2	3
